# 3,3′,5,5′-Tetra­bromo-2,2′-bithio­phene

**DOI:** 10.1107/S1600536809011647

**Published:** 2009-04-02

**Authors:** Hongqi Li, Lin Li

**Affiliations:** a Key Laboratory of Science & Technology of Eco-Textiles, Ministry of Education, College of Chemistry, Chemical Engineering & Biotechnology, Donghua University, Shanghai 201620, People’s Republic of China

## Abstract

The title compound, C_8_H_2_Br_4_S_2_, was prepared by bromination of 2,2′-bithio­phene with bromine. The mol­ecule is located on a crystallographic twofold rotation axis, thereby imposing equal geometry of the two thio­phene rings. Each five-membered ring is planar [maximum deviation 0.011 (9) Å] and the dihedral angle between the planes through the rings is 47.2 (4)°. The mol­ecules are arranged to minimize intramolecular contacts between the 3-3′ and 5-5′-bromine atoms.

## Related literature

For use of the title compound as an intermediate in the synthesis of oligothiophenes and polythiophenes, see: Roncali (1997 ▶); Funahashi *et al.* (2005 ▶). For synthetic methods, see: Takahashi *et al.* (2006 ▶); Lin *et al.* (2005 ▶).
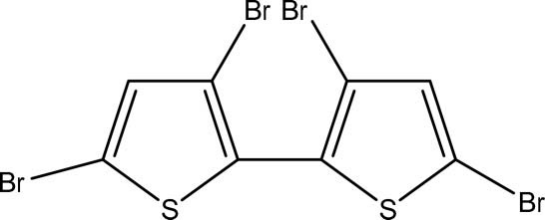


## Experimental

### 

#### Crystal data

C_8_H_2_Br_4_S_2_
*M* *_r_* = 481.86
Monoclinic, 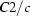

*a* = 17.164 (3) Å
*b* = 4.0153 (7) Å
*c* = 18.655 (3) Å
β = 115.395 (3)°
*V* = 1161.4 (4) Å^3^
*Z* = 4
Mo *K*α radiation
μ = 14.18 mm^−1^
*T* = 293 K
0.40 × 0.17 × 0.05 mm

#### Data collection

Bruker SMART CCD area-detector diffractometer
Absorption correction: multi-scan (*SADABS*; Sheldrick, 2004 ▶) *T*_min_ = 0.258, *T*_max_ = 1.000 (expected range = 0.122–0.472)
2792 measured reflections
1077 independent reflections
886 reflections with *I* > 2σ(*I*)
*R*_int_ = 0.146

#### Refinement

*R*[*F* ^2^ > 2σ(*F* ^2^)] = 0.078
*wR*(*F* ^2^) = 0.208
*S* = 1.00
1077 reflections
64 parameters
H-atom parameters constrained
Δρ_max_ = 1.15 e Å^−3^
Δρ_min_ = −1.06 e Å^−3^

### 

Data collection: *SMART* (Bruker, 2001 ▶); cell refinement: *SAINT* (Bruker, 2001 ▶); data reduction: *SAINT*; program(s) used to solve structure: *SHELXS97* (Sheldrick, 2008 ▶); program(s) used to refine structure: *SHELXL97* (Sheldrick, 2008 ▶); molecular graphics: *SHELXTL* (Sheldrick, 2008 ▶); software used to prepare material for publication: *SHELXTL*.

## Supplementary Material

Crystal structure: contains datablocks global, I. DOI: 10.1107/S1600536809011647/kj2109sup1.cif

Structure factors: contains datablocks I. DOI: 10.1107/S1600536809011647/kj2109Isup2.hkl

Additional supplementary materials:  crystallographic information; 3D view; checkCIF report

## Figures and Tables

**Fig. 1.**
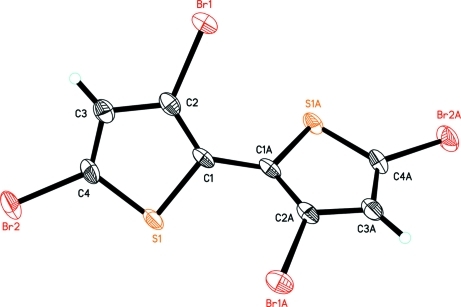
A view of the molecule of the title compound. Displacement ellipsoids are drawn at the 30% probability level.

## supplementary crystallographic information

### Comment

3,3',5,5'-Tetrabromo-2,2'-bithiophene is an important intermediate compound in the synthesis of oligothiophenes and polythiophenes which have recently attracted attention as materials showing conductive, semiconductive, nonlinear optical (NLO), and liquid crystalline characteristics (Roncali, 1997; Funahashi *et al.*, 2005). While synthesis of 3,3',5,5'-tetrabromo-2,2'-bithiophene could be achieved by coupling of 2,3-dibromothiophene (Takahashi *et al.*, 2006) or bromination of 2,2'-bithiophene (Lin *et al.*,  2005), its single crystal structure has not been reported. Herein we present the single crystal structure of the title compound. A molecule of the title compound is located on a crystallographic two-fold rotation axis, thereby imposing equal geometry of the two rings. Each 5-membered ring is planar and the dihedral angle between the planes thorugh the rings is 47.2 (4)°. The molecules arrange in such a fashion that both pairs of bromine atoms (3- and 3'-bromine and 5- and 5'-bromine) lie far away to each other.

### Experimental

The title compound was prepared as reported in the literature (Lin *et al.*, 2005). Single crystals suitable for X-ray diffraction measurement were obtained by slow evaporation of a solution in ethanol (m.p. 413 K; literature value: 413–414 K (Takahashi *et al.*, 2006)).

### Refinement

All H atoms were placed at calculated positions and refined using a riding model approximation, with C—H = 0.93 Å and with *U*_iso_(H) = 1.2*U*_eq_(C).

### Crystal data

**Table:**

C_8_H_2_Br_4_S_2_	*D*_x_ = 2.756 Mg m^−^^3^
*M**_r_* = 481.86	Melting point = 413–414 K
Monoclinic, *C*2/*c*	Mo *K*α radiation, λ = 0.71073 Å
*a* = 17.164 (3) Å	Cell parameters from 1249 reflections
*b* = 4.0153 (7) Å	θ = 4.8–55.3°
*c* = 18.655 (3) Å	µ = 14.18 mm^−^^1^
β = 115.395 (3)°	*T* = 293 K
*V* = 1161.4 (4) Å^3^	Prismatic, yellow
*Z* = 4	0.40 × 0.17 × 0.05 mm
*F*(000) = 888	

### Data collection

**Table:**

Bruker SMART CCD area-detector diffractometer	1077 independent reflections
Radiation source: fine-focus sealed tube	886 reflections with *I* > 2σ(*I*)
graphite	*R*_int_ = 0.146
φ and ω scans	θ_max_ = 25.5°, θ_min_ = 2.4°
Absorption correction: multi-scan (*SADABS*; Sheldrick, 2004)	*h* = −20→18
*T*_min_ = 0.258, *T*_max_ = 1.000	*k* = −4→4
2792 measured reflections	*l* = −22→21

### Refinement

**Table:**

Refinement on *F*^2^	Primary atom site location: structure-invariant direct methods
Least-squares matrix: full	Secondary atom site location: difference Fourier map
*R*[*F*^2^ > 2σ(*F*^2^)] = 0.078	Hydrogen site location: inferred from neighbouring sites
*wR*(*F*^2^) = 0.208	H-atom parameters constrained
*S* = 1.00	*w* = 1/[σ^2^(*F*_o_^2^) + (0.1428*P*)^2^] where *P* = (*F*_o_^2^ + 2*F*_c_^2^)/3
1077 reflections	(Δ/σ)_max_ < 0.001
64 parameters	Δρ_max_ = 1.15 e Å^−^^3^
0 restraints	Δρ_min_ = −1.06 e Å^−^^3^

### Special details

**Table:**

Geometry. All e.s.d.'s (except the e.s.d. in the dihedral angle between two l.s. planes) are estimated using the full covariance matrix. The cell e.s.d.'s are taken into account individually in the estimation of e.s.d.'s in distances, angles and torsion angles; correlations between e.s.d.'s in cell parameters are only used when they are defined by crystal symmetry. An approximate (isotropic) treatment of cell e.s.d.'s is used for estimating e.s.d.'s involving l.s. planes.
Refinement. Refinement of *F*^2^ against ALL reflections. The weighted *R*-factor *wR* and goodness of fit *S* are based on *F*^2^, conventional *R*-factors *R* are based on *F*, with *F* set to zero for negative *F*^2^. The threshold expression of *F*^2^ > σ(*F*^2^) is used only for calculating *R*-factors(gt) *etc*. and is not relevant to the choice of reflections for refinement. *R*-factors based on *F*^2^ are statistically about twice as large as those based on *F*, and *R*- factors based on ALL data will be even larger.

### Fractional atomic coordinates and isotropic or equivalent isotropic displacement parameters (Å^2^)

**Table:**

	*x*	*y*	*z*	*U*_iso_*/*U*_eq_	
Br1	0.17755 (6)	0.2991 (3)	0.27753 (6)	0.0443 (5)	
Br2	0.11718 (8)	0.7463 (3)	0.54198 (6)	0.0562 (5)	
S1	−0.00225 (17)	0.7354 (6)	0.36201 (13)	0.0393 (7)	
C1	0.0301 (5)	0.581 (2)	0.2918 (4)	0.0331 (17)	
C2	0.1140 (5)	0.480 (2)	0.3291 (4)	0.0354 (17)	
C4	0.0973 (6)	0.650 (2)	0.4373 (5)	0.041 (2)	
C3	0.1540 (5)	0.521 (2)	0.4129 (4)	0.0411 (19)	
H3	0.2109	0.4669	0.4459	0.049*	

### Atomic displacement parameters (Å^2^)

**Table:**

	*U*^11^	*U*^22^	*U*^33^	*U*^12^	*U*^13^	*U*^23^
Br1	0.0524 (7)	0.0457 (7)	0.0483 (7)	0.0023 (4)	0.0344 (6)	−0.0050 (4)
Br2	0.0735 (9)	0.0718 (9)	0.0312 (7)	−0.0072 (5)	0.0298 (6)	−0.0063 (4)
S1	0.0508 (15)	0.0482 (13)	0.0306 (12)	0.0043 (9)	0.0286 (11)	−0.0007 (8)
C1	0.050 (5)	0.030 (4)	0.034 (4)	0.000 (4)	0.032 (4)	0.000 (3)
C2	0.052 (5)	0.030 (4)	0.034 (4)	−0.006 (3)	0.028 (4)	0.001 (3)
C4	0.059 (6)	0.042 (5)	0.028 (4)	0.003 (4)	0.025 (4)	0.006 (3)
C3	0.050 (5)	0.046 (5)	0.035 (4)	−0.003 (4)	0.025 (4)	0.005 (4)

### Geometric parameters (Å, °)

**Table:**

Br1—C2	1.882 (8)	C1—C1^i^	1.455 (15)
Br2—C4	1.873 (8)	C2—C3	1.422 (11)
S1—C4	1.719 (9)	C4—C3	1.342 (11)
S1—C1	1.741 (7)	C3—H3	0.9300
C1—C2	1.365 (11)		
			
C4—S1—C1	91.0 (4)	C3—C4—S1	114.3 (6)
C2—C1—C1^i^	131.0 (8)	C3—C4—Br2	126.8 (7)
C2—C1—S1	109.2 (6)	S1—C4—Br2	118.9 (5)
C1^i^—C1—S1	119.8 (7)	C4—C3—C2	109.8 (8)
C1—C2—C3	115.6 (7)	C4—C3—H3	125.1
C1—C2—Br1	124.7 (6)	C2—C3—H3	125.1
C3—C2—Br1	119.6 (6)		
			
C4—S1—C1—C2	−0.9 (6)	C1—S1—C4—C3	1.7 (7)
C4—S1—C1—C1^i^	179.8 (5)	C1—S1—C4—Br2	−179.2 (5)
C1^i^—C1—C2—C3	179.2 (5)	S1—C4—C3—C2	−1.9 (10)
S1—C1—C2—C3	0.1 (9)	Br2—C4—C3—C2	179.1 (6)
C1^i^—C1—C2—Br1	−0.2 (11)	C1—C2—C3—C4	1.2 (11)
S1—C1—C2—Br1	−179.4 (4)	Br1—C2—C3—C4	−179.4 (6)

Symmetry codes: (i) −*x*, *y*, −*z*+1/2.
